# Induced Pluripotent Stem Cell Potential in Medicine, Specifically Focused on Reproductive Medicine

**DOI:** 10.3389/fsurg.2014.00005

**Published:** 2014-03-24

**Authors:** Olivier Botman, Christine Wyns

**Affiliations:** ^1^Gynecology Unit, Medical School, Institut de Recherche Expérimentale et Clinique, Université Catholique de Louvain, Brussels, Belgium; ^2^Cliniques Universitaires Saint-Luc, Université Catholique de Louvain, Brussels, Belgium

**Keywords:** human induced pluripotent stem cells, primordial germ cells, human germ cell differentiation, embryonic cells, infertility

## Abstract

Since 2006, several laboratories have proved that somatic cells can be reprogramed into induced pluripotent stem cells (iPSCs). iPSCs have enormous potential in stem cell biology as they can give rise to numerous cell lineages, including the three germ layers. In this review, we discuss past and recent advances in human iPSCs used for modeling diseases *in vitro*, screening drugs to test new treatments, and autologous cell and tissue regenerative therapies, with a special focus on reproductive medicine applications. While this latter field of research is still in its infancy, it holds great promise for investigating germ cell development and studying the genetic and physiopathological mechanisms of infertility. A major cause of infertility is the absence of germ cells in the testes, mainly due to genetic background or as a consequence of gonadotoxic treatments. For these patients, no effective fertility restoration strategy has so far been identified. The derivation of germ cells from iPSCs represents an alternative source of stem cells able to differentiate into spermatozoa. Lessons learned from animal models as well as studies on human iPSCs for reproductive purposes are reviewed.

## Introduction

By definition, stem cells can differentiate spontaneously into all cell types that form the human body. They have the ability to both differentiate into other mature cell types and maintain an undifferentiated state by self-renewal. These unique properties form the basis for stem cell use in cell and tissue regeneration. Currently, embryonic stem cells (ESCs) are the most widely studied stem cell type. ESCs arise from culture of primitive ectoderm cells of the inner cell mass of blastocysts and show pluripotency properties. Under strict culture conditions, they can perpetuate their undifferentiated pluripotent state indefinitely and are therefore an *in vitro* derivative without a specific *in vivo* counterpart. Since the first human ESC (hESC) line was obtained in 1998 by Thomson (1), numerous hESC lines have been recorded in the hESC registry [www.hESCreg.eu (2)]. However, some ethical issues regarding the use and destruction of human embryos, as well as concerns about genetic identity or immunological rejection by the recipient (3, 4), represent serious limitations for hESC application in humans. Obtaining pluripotent stem cells from alternative sources such as adult somatic cells, known as induced pluripotent stem cells (iPSCs), has therefore been contemplated. The aim of this review is to present their current applications and investigate their potential use in clinical practice in the light of animal studies.

The first iPSC lines were generated from adult fibroblasts by retrovirus-mediated introduction of four transcription factors into the genome of somatic cells (*OCT4, SOX2, C-MYC*, and *KLF4*) (5). *OCT4* (6) and *SOX2* (7) are core transcription factors of pluripotency, while *C-MYC* (8) and *KLF4* (9) are involved in self-renewal. Retroviruses appear to be required only for induction of pluripotency and not for its maintenance, as demonstrated after strong silencing of the four retroviruses (10). Epigenetic reprograming of autologous somatic cells into PSCs has attracted much attention because of the potential for autotransplantation therapy, as cellular derivatives of reprogramed cells will not be rejected by the recipient and there are no ethical concerns as for embryonic cells. iPSCs have been shown to be equivalent to ESCs in terms of morphology, surface markers, gene expression, proliferation capacity, and differentiation (11). Indeed, karyotype analysis revealed no notable difference in the incidence of chromosomal aberrations in iPSCs compared to hESCs (12). Although reprograming occurs at a very low frequency and with only partial epigenetic reprograming, as determined by the methylated status of *OCT4* in iPSCs cells (13), it appears to be sufficient to generate reprogramed cell lines that can be used *in vitro* indefinitely.

## Materials and Methods

We conducted an extensive Medline search using the following search terms: iPSCs and germ cell differentiation. A total of 5897 articles dating from 1967 to 2013 were initially retrieved. Since the topic is innovative, original articles of any design and review articles published in English and French were suitable for inclusion. Selection criteria were based on the main outcome of interest referenced in this baseline of articles, namely the potential in reproductive medicine of iPSCs reprogramed from animal and human somatic cells, including differentiation into germ lines and infertility modeling, with a view to synthesizing the state of current knowledge for clinical applicability in humans. Regarding issues connected to the main subject, namely use of iPSC line differentiation to (a) understand the physiopathology of diseases, (b) study the efficacy and toxicology of new medical therapy, and (c) regenerate cells and tissues, the goal was to introduce the reader to the literature, rather than provide an exhaustive review. The final number of studies referenced in this review is 135.

### iPSCs used to study/understand diseases

Since the creation of the first-line of iPSCs from mature adult cells by Takahashi and Yamanaka (5), generating patient-specific stem cells through reprograming has become almost routine. iPSC technology provides a uniquely useful disease-specific tool to analyze normal development, outline disease features, and study the physiopathological and genetic mechanisms of the disease *in vitro*. With the increase in stem cell line collection during the past few years, disease models have been created from human adult somatic cells by reprograming. Although iPSCs can be patently derived from any type of somatic cells, they are mostly reprogramed from skin fibroblasts, due to ease of accessibility. Table 1 summarizes fully differentiated disease-specific iPSC lines obtained from somatic cells so far, as well as iPSCs used for physiopathological screening and drug testing (Table A1 in Appendix).

**Table 1:** **Differentiation of human iPSCs into male germ cell lineage**.

Reference	Cell source	Additional factors	*In vitro*-derived cells	Main evaluation
(42)	Fibroblast XY	Fetal gonadal cells	PGCs	Expression (STELLA, VASA, ACROSIN)
				Genomic imprint (*H19, PEG1*)
(31)	Fibroblast XY, XX	RA, forskolin, and CYP26	PGCs spermatids	Expression (VASA, SYCP3)
				Genomic imprint (*H19*)
				Genome ploidy
(40)	Fibroblast XY, XX	BMP 4, 7, and 8	Spermatids	Expression (VASA, ACROSIN)
		*DAZ* family overexpression		Genome ploidy
(39)	Fibroblast XY, XX	*VASA/DAZL* overexpression	Spermatids	Expression (VASA, ACROSIN)
				Genomic imprint (*H19*)
				Genome ploidy
(43)	Fibroblast XX, XY, XXY	BMP 4, 7, and 8	PGCs	XCI (*H3K27me3, macroH2A1*)
				Transcriptome of differentially expressed X-linked genes
(38)	Fibroblast XY	BMP 4, 7, and 8	SSCs spermatocytes	Expression (VASA, ACROSIN)
			Spermatids	Genomic imprint (*H19, IGF2*)
				Genome ploidy

*RA, retinoic acid; CYP26, cytochrome P26; BMP, bone morphogenic protein; XCI, X chromosome inactivation; DEG, differentially expressed gene; SSC, spermatogonial stem cell*.

### iPSCs: Toxicology studies and new medical treatments

Induced pluripotent stem cells technology provides a unique platform to identify possible therapeutic agents, evaluate their efficacy and toxicity, and study gene repair associated with cell replacement therapy. Indeed, derivation of patient-specific familial dysautonomia (FD) iPSCs (14) illustrates the potential of iPSC technologies for modeling therapeutic action in human disease *in vitro*. FD is a peripheral neuropathy caused by a point mutation in the IKBKAP8 gene, characterized by depletion of autonomic and sensory neurons. After differentiation of FD iPSCs into peripheral neurons, the effect of candidate drugs in reversing aberrant splicing and improving neuronal differentiation and migration may be studied. In addition, while kinetin was reported to affect splicing and absolute levels of IKBKAP8 (15), exposure of FD-iPSC-derived neural crest precursors to kinetin was shown to result in a dramatic reduction in the mutant IKBKAP8 splice form and a significant increase in the percentage of differentiating neurons under continuous kinetin treatment, demonstrating the potential usefulness of disease-specific iPSCs in developing new drug therapies.

Use of cardiomyocytes differentiated from human catecholaminergic polymorphic ventricular tachycardia (CPVT) iPSCs has provided insights into arrhythmia mechanisms in CPVT, a calcium-dependent familial arrhythmogenic disorder associated with dominant mutations in the cardiac ryanodine receptor gene, allowing screening of the effects of disease aggravators (adrenergic stimulation) and drug treatments (beta blockers and flecainide) (16). Analysis of the iPSC line showed mutation into the ryanodine receptor gene to be linked with altered calcium release, and found tested treatments to be effective *in vitro*.

These latter developments demonstrate the feasibility of using *in vitro* iPSC differentiation assays for drug testing, providing a unique tool in the presence of *in vivo* study limitations in humans. Thus, human iPSCs may be used for personalized medicine, with pharmacological and toxicological tests designed and performed on an individual’s genome.

### iPSCs for cell/tissue regenerative therapy: From animal to human application

Beyond *in vitro* use of human iPSC lines, clinical application of iPSC therapies seems rather unrealistic (see iPSCs Differentiation into Male Gamete Lineage). However, a number of studies in animals have opened new perspectives for human therapeutic applications. In 2007, Hanna and colleagues treated a humanized mouse model of sickle-cell anemia by transplantation of iPSCs derived from mouse skin cells repaired with a homologous recombination. Transplanting these repaired iPSCs differentiated into hematopoietic progenitors led to correction of the disease phenotype in the sick mice (17). In 2008, Wernig derived dopaminergic neurons from iPSCs and found, after engrafting into the brain, that they survived, were functional and able to partially rescue a rat model of Parkinson’s disease (18, 19). These two studies, showing stable and functional engraftment of repaired specific iPSCs, demonstrate the huge potential of iPSC-based treatment. Moreover, human iPSCs have already shown beneficial effects after their differentiation and transplantation into mouse-specific disease models. Transplantation of human iPSCs into the subretinal space of a mouse model of retinitis pigmentosa after differentiation into functional retinal pigmented epithelial tissue showed stable long-term engraftment, assimilation into the host retina without disruption, and improved visual function over the lifetime (20). These results, and the absence of tumor development in transplanted mice, suggest that such therapies would be transposable to human clinical practice and would improve classical treatment.

In humans, a number of clinical studies have already revealed the benefits of autologous non-iPSC transplantation, particularly for functional recovery (21–23). While use of iPSCs as a source for autologous stem cell transplantation is still in its infancy, some iPSC applications in humans are well on the way to being introduced into a clinical setting. Indeed, insulin-producing cells derived from human iPSCs have already been obtained *in vitro* for potential therapeutic use in diabetes (24).

### iPSCs and reproduction

There is no doubt that parenthood plays an important role in quality of life, so fertility preservation or restoration strategies need to be developed for infertile patients. Storage of spermatozoa, oocytes, or ovarian tissue should be the first-line treatment approach, as their reproductive potential after freezing has already been proven in humans. However, storage of mature or immature germ cells is not always possible, either because of lack of time in an emergency context like cancer therapy, or inaccessibility to the technique at the time of disease management. For these patients, generation of gametes from iPSCs would be an innovative strategy that could give them hope of becoming parents. Use of non-gametogenic pluripotent stem cells as a source of germ cells could also benefit patients suffering from congenital diseases affecting reproduction, such as Klinefelter syndrome, Y chromosome microdeletions, and Turner syndrome, who may have already lost their germ cells at the time of diagnosis. It could also be effective for patients whose germ cells are not functioning, for instance boys with cryptorchid testes.

During embryonic development, primordial germ cells (PGCs), differentiated from epiblast cells, are identifiable at 4 weeks of gestation and migrate through the epiblastic crest to colonize the gonadal ridges by 7 weeks of gestation (25, 26). While migrating, PGCs proliferate intensively and begin extensive nuclear reprograming to regain self-renewal capacities and reset their genomic imprinting. Germ cells are highly specialized cells established by a specific transcriptional program, including repression of somatic fate and regulation of the extensive epigenetic reprograming of the genome (27). Cells that undergo differentiation into PGCs show expression of some key pluripotency-specific genes that appear to play a role in germ cell specification in mammals, such as Blimp 1 (or Prdm1) (28) and Prdm 14 (29). These processes are completed after reaching the gonadal ridge. After some rounds of proliferation, PGCs finally differentiate into oogonia or gonocytes within the sex cords for female and male individuals respectively.

Due to the complexity of gametogenesis *in vivo*, mimicking germ cell differentiation *in vitro* will help us better understand the regulation of developmental programs, such as specification, migration, and sex determination, which allow transmission of genetic information and creation of new human beings. The capacity of iPSCs to differentiate into germ cells of both genders has been tested in several species, and recent studies have demonstrated that PGCs can be obtained by *in vitro* differentiation of iPSCs, producing functional gametes and offspring in mice (10). In an attempt to shed new light on the benefits of using iPSCs in reproductive medicine, this review focuses on results obtained from differentiation of mouse and human iPSCs into germ cell lineage.

### iPSCs differentiation into male gamete lineage

#### Lessons learned from animal models

Spontaneous differentiation of iPSCs occurs after 4–7 days of culture and is highly variable and inconstant, resulting in different cell types from the three germ layers (endoderm, mesoderm, and ectoderm) in varying amounts. Several studies have reported derivation of germ cell precursors and gametes from mouse iPSCs. Injection of iPSCs into blastocysts generated chimeric pups, and analysis of host organs demonstrated the extensive contribution of injected iPSCs to various organs, including the eyes, ears, tail, claws, kidneys, liver, lungs, stomach, guts, and testes (30), confirming the potential of iPSCs to form gametes *in vivo*. Differentiation of iPSCs to PGCs occurs *in vitro* when factors that promote self-renewal, such as feeder cells and βFGF, are removed from the culture medium (31, 32), albeit at a low frequency. Research should therefore focus on selection and enrichment of specific cell lineages toward directed differentiation. Improvement of germ cell differentiation, evidenced by enhanced expression of pre-meiotic and meiotic germ cell-specific genes, was observed in iPSCs derived from embryoid bodies (EBs) (33). Selection of germ cells from these EBs may be achieved by a simple density gradient procedure (34). Furthermore, injection of testicular cells and iPSCs into the dorsal skin of mice led to reconstitution of seminiferous tubules, with iPSC-derived germ cells settling on basement membranes of reconstituted tubules (33, 35). While iPSCs are most commonly derived from skin fibroblasts, iPSCs derived from adult mouse hepatocytes can also give rise to presumptive germ cells (36). iPSCs can therefore produce candidate male germ cells *in vitro*, independently of their origin. Moreover, iPSCs transplanted into the seminiferous tubules of W/W^v^ mice, lacking endogenous spermatogenesis, are able to undergo their own spermatogenesis and generate offspring after ICSI followed by embryo transfer (37). Although most transplanted mice died and some pups presented with tumors, this study demonstrates the potential of germ-like cells in fertility recovery.

#### Studies on human iPSCs

During spontaneous iPSC differentiation, a small population of male germ cells, including round spermatid-like cells, was observed (38, 39), suggesting the possibility of achieving differentiation and maturation of iPSCs into spermatozoa. Panula (40) showed that human iPSCs grown in a medium enriched with bone morphogenic protein 4 (BMP4) can differentiate into PGCs, albeit at a low efficiency of just 5%. The deleted in azoospermia (DAZ) gene family and human deleted in azoospermia-like (DAZL) are involved in PGC formation, whereas the Y chromosome homolog DAZ and closely related BOULE promote later stages of meiosis and development of haploid gametes (41). Indeed, meiosis was entered when DAZ family proteins (DAZL, BOULE, and DAZ) were overexpressed and some PGCs continued their maturation into haploid cells, showing an acrosomal complex identical to that of spermatids (40). Through similar experiments, Eguizabal et al. (31) obtained haploid cells using a medium enriched with retinoic acid, a differentiating factor acting as a trigger for meiosis. Haploid male germ line acrosin-positive cells were consistently obtained, without overexpression of any developmentally related genes, from human iPSCs of different origin (fibroblasts, keratinocytes, or cord blood), suggesting independence from the epigenetic memory of reprogramed somatic cells.

So far, no mature sperm have been obtained *in vitro* and there are still many unknowns concerning autocrine, paracrine, and endocrine hormonal factors, as well as the nutritional control of germ cell maturation. In this regard, attempts to differentiate germ cells from human iPSCs, with key factors used to direct them into germ cell lineage, are summarized in Table 1.

### iPSC differentiation into female gamete lineage

While numerous studies have demonstrated the ability of ESCs to differentiate into female germ cells, with some groups reporting formation of follicle-like structures and oocyte-like cells (44–53), only three studies in animals have investigated the potential of iPSCs to differentiate into female germ cell lineage. Among germ cells derived from iPSCs, several have shown the potential to differentiate into oocyte-like cells. When iPSCs from male or female animals were cultured in EB formations, some round-shaped cells were found to express mouse vasa homolog gene (Mvh), an early PGC reporter. In addition, these round-shaped cells showed expression of early oocyte-like markers (54), demonstrating the capacity of iPSCs to differentiate into female germ cell lineage. The ability of iPSCs to differentiate into oogonia was evidenced by expression of oocyte markers Zp2 and Zp3 after exposure of iPSCs to RA, although at a smaller proportion than in differentiation into spermatogonial lineage (55, 56).

### Technical limitations

Since the discovery of PSCs in human beings, scientists have looked at the possibility of using this source of special cells to regenerate tissue and organs, with a considerably reduced risk of an immune response. Despite the great promise of iPSC technology, there are still barriers to overcome before these cells can be used in a clinical context.

The gold standard technique utilized to reprogram somatic cells is the inducible lentiviral vector that reaches 2% efficiency (57). While it shows relatively good efficacy, the lentiviral vector requires genomic integration to reprogram somatic cells. Retroviral vectors contain transcription factors that are potentially oncogenic, especially oncogene *c-MYC*, although it has been shown to be dispensable for iPSC generation (18). However, exogenous *OCT4* (58), *KLF4* (59), and *NANOG* (60) can also cause teratoma formation. Indeed transplantation of a single undifferentiated cell might result in tumor formation or proliferation of inappropriate cell types. Retroviral vectors are silenced after reprograming, but slight reactivation has been observed during differentiation, although this did not appear to have a major impact on germline-directed differentiation of iPSCs. Unfortunately, selection of iPSCs with a low viral copy number is insufficient to eliminate the oncogenic risk. Besides tumor formation, there is a risk of genetic recombination or insertion mutagenesis, which can affect cell differentiation due to random vector integration into the genome. Even for *in vitro* applications of iPSCs, such as disease modeling, drug screening, or toxicology tests, re-expression of exogenous factors resulting in genome modification could disturb the properties of cells and yield biased results. Therefore, production of iPSCs with minimal or no genetic modifications is essential. Maintenance of a stable karyotype (61) and elimination of the risk of tumor formation required for clinical use of iPSC lines are challenging areas in iPSC technology.

### Strategies to ensure safety aspects

Induced pluripotent stem cell lines carry the risk of mutagenesis. Strategies to overcome this barrier and eventually offer the possibility of potential application in humans should be implemented. Excisable Lentiviral (62) and transposon (63) vectors deliver reasonably good reprograming efficiency (0.1–1%), but require constant and intensive screening of excised cell lines. Other non-integrating methods, such as repeat application of plasmid (64), episomal, or adenovirus (65) vectors with transient expression, have the disadvantage of low efficiency (0.001%) and occasional genomic integration. The most recent technologies employ DNA-free methods to reprogram cells. Use of Sendai virus (66), modified mRNA (67), micro-RNA (68), or proteins (69) that modulate the reprograming process could be a powerful approach to generate more efficient and safer iPSCs. The modified mRNA method in particular shows encouraging results, with efficacy reaching 4.4%. In addition, modified mRNAs bypass innate antiviral responses, have a fast kinetic response and are applicable to a range of tissue engineering tasks (68).

Finally, reprograming technology allows avoidance of the stem cell stage by direct reprograming, converting endogenous cells directly into desired cell types by gene transfer of defined factors, as has been demonstrated in hepatocyte (70), neuron (71), and cardiomyocyte differentiation (72). Direct reprograming has the principal advantage of drastically reducing the risk of contamination with undifferentiated cells, and hence the risk of transplanting those cells (73, 74). While there appears to be a consensus on the need to exclude undifferentiated cells, the level of differentiation required for clinical use of iPSCs is still an open question.

## Conclusion

Numerous pre-clinical trials (shown in Table A1 in Appendix) have convincingly shown that iPSCs can be differentiated into cells with the capacity for tissue or cell repair, but there is still a long way to go before all differentiation issues are adequately addressed. Besides demonstrating full and safe functioning, development of clinical-grade iPSCs has to meet the safety requirements of regulatory bodies, namely being virus integration-free (62), respecting xeno free conditions (75), using of a synthetic matrix (76), and ultimately applying GMP-compliant reprograming technology. Meanwhile, iPSCs provide a useful platform for understanding diseases and establishing the efficacy and toxicity of new therapies. In reproductive medicine, they represent a tool to study human germ cell development and fertility defects, and offer perspectives to restore fertility in patients presenting with irreversible infertility due to gonadotoxic treatment or genetic background. As such, iPSCs show great promise and will undoubtedly be the subject of active research in the coming years.

## Conflict of Interest Statement

The authors declare that the research was conducted in the absence of any commercial or financial relationships that could be construed as a potential conflict of interest.

## Appendix

**Table A1:** **Diseases modeled with fully differentiated disease-specific iPSCs**.

Disease models	Reference	Somatic cell source	Physiopathological screening or drug testing
**METABOLIC DISEASES**			
Lesch–Nyhan syndrome (carrier)	(77)	Fibroblast	N
Gaucher’s disease, type III	(78)	Fibroblast	Y
Type 1 diabetes	(79)	Fibroblast	N
α1-Antitrypsin deficiency	(80)	Fibroblast	N
	(81)	Fibroblast	N
Glycogen storage disease Ia	(81)	Fibroblast	N
	(82)	Fibroblast	Y
Familial hypercholesterolemia	(81)	Fibroblast	N
Crigler–Najjar syndrome	(81)	Fibroblast	N
	(82)	Fibroblast	Y
Hereditary tyrosinemia, type 1	(81)	Fibroblast	N
	(82)	Fibroblast	Y
Hurler syndrome	(83)	Fibroblast	N
		Keratinocyte	
Mucopolysaccharidosis type IIIB	(84)	Fibroblast	Y
Niemann-Pick type C1	(85)	Fibroblast	N
**NEUROLOGICAL DISEASES**			
Parkinson’s disease	(86)	Fetal cortical progenitor	Y
	(77)	Fibroblast	N
	(87)	Fibroblast	Y
	(88)	Fetal lung fibroblast	Y
		Bone marrow mesenchymal stem cells	
	(89)	Fibroblast	Y
	(90)	Fibroblast	N
	(91)	Fibroblast	Y
Huntington’s disease	(92)	Fibroblast	N
	(93)	Fibroblast	N
Familial amyotrophic lateral sclerosis	(94)	Fibroblast	N
	(95)	Fibroblast	Y
Familial dysautonomia	(14)	Fibroblast	Y
Rett syndrome	(96)	Fibroblast	Y
	(97)	Fibroblast	N
	(98)	Fibroblast	N
Spinal muscular atrophy	(99)	Fibroblast	Y
	(100)	Fibroblast	N
Angelman’s syndrome	(101)	Fibroblast	Y
Prader–Willi syndrome	(101)	Fibroblast	N
	(102)	Fibroblast	N
Friedriech’s ataxia	(103)	Fibroblast	N
Schizophrenia	(104)	Fibroblast	Y
Machado–Joseph disease	(105)	Fibroblast	Y
Childhood cerebral Adrenoleukodystrophy and adrenomyeloneuropathy	(106)	Fibroblast	Y
Alzheimer’s disease	(107)	Fibroblast	Y
	(108)	Fibroblast	Y
Warkany syndrome 2 X-linked adrenoleukodystrophy	(106)	Fibroblast	Y
	(20)	Amniocyte	Y
		Fibroblast	
Emanuel syndrome	(20)	Amniocyte	N
		Fibroblast	
**RETINOPATHIES**			
Gyrate atrophy	(109)	Fibroblast	Y
Retinitis pigmentosa	(110)	Fibroblast	N
	(111)	Fibroblast	Y
	(132)	Fibroblast	Y
Leber’s congenital amaurosis	(112)	Fibroblast	N
Usher syndrome			N
Leber’s hereditary optic neuropathy			N
**IMMUNE AND BLOOD DISEASES**			
Fanconi’s anemia	(113)	Fibroblast	N
β-Thalassemia	(134)	Fibroblast	N
Polycythemia vera	(134)	CD34+ cell	N
Primary myelofibrosis	(134)		N
Sickle-cell anemia	(80)	Fibroblast	N
Scleroderma		Fibroblast	N
Chronic myeloid leukemia disease	(115)	Fibroblast	N
	(116)	CD34+ cell	N
Severe congenital neutropenia	(117)	Fibroblast	N
**CARDIOVASCULAR DISEASES**			
LEOPARD syndrome	(118)	Fibroblast	N
Long-QT 1	(119)	Fibroblast	N
Timothy syndrome	(120)	Fibroblast	Y
Overlapping Na^+^ channel disease syndrome	(121)	Fibroblast	N
Familial dilated cardiomyopathy	(122)	Fibroblast	Y
Long-QT 2	(123)	Fibroblast	Y
	(124)	Fibroblast	Y
	(125)	Fibroblast	Y
Catecholaminergic polymorphic ventricular tachycardia	(126)	Fibroblast	Y
	(127)	Fibroblast	Y
	(128)	Fibroblast	Y
	(16)	Fibroblast	Y
	(129)	Fibroblast	Y
Arrhythmogenic right ventricular cardiomyopathy	(133)	Fibroblast	N
	(130)	Fibroblast	Y
**OTHER DISEASES**			
Down syndrome	(131)	Fibroblast	N
Cystic fibrosis	(80)	Fibroblast	N
Recessive dystrophic epidermolysis bullosa	(83)	Fibroblast	N
		Keratinocyte	
Patau syndrome	(20)	Amniocyte	Y
		Fibroblast	
Klinefelter syndrome	(43)	Fibroblast	N
